# Applicability analysis of flame height estimation based on Byram’s fireline intensity model under flat and windless conditions

**DOI:** 10.1038/s41598-024-55132-3

**Published:** 2024-02-23

**Authors:** Yunlin Zhang, Aixia Luo

**Affiliations:** 1 School of Biological, Guizhou Education University, Gaoxin St. 115, Guiyang, 550018 China; 2 Key Laboratory of Ecology and Management on Forest Fire in Universities of Guizhou Province, Guizhou Education University, Gaoxin St. 115, Guiyang, 550018 China

**Keywords:** Flame height, Fire intensity, Yunnan–Guizhou Plateau, Prediction model, Forest fire, Fire behavior, Fire ecology, Forestry

## Abstract

Forest fire have a serious impact on forest ecosystems, the safety of people’s lives and property, and social stability. The height of surface flames, as the main indicator of forest fire behavior, which is an important parameter for forest fire management. The relationship between fireline intensity and flame height proposed by Byram has been widely used in estimating flame height; however, its applicability to the surface fuel of typical forest stands in the Yunnan–Guizhou Plateau of China has not yet been analyzed. In this study, the surface fuel in the area was taken as the research object, and the flame height of different fuel bed characteristics was measured through an indoor burning experiment. The applicability of three methods—the directly used Byram’s model, corrected model, and re-established prediction model—was analyzed to estimate the flame height in the Yunnan–Guizhou Plateau. We found that the flame height of the typical forest stands in the Yunnan–Guizhou Plateau ranged from 0.05 to 1.2 m and was significantly affected by the moisture content, load, and height of the fuel bed. Although the fireline intensity exhibited a significant linear relationship with the flame height, directly using Byram’s method to predict the flame height of surface fires was impractical, as its mean prediction error exceeded 150%. The mean relative errors of the prediction model obtained by modifying Byram’s method and that based on the characteristics of the fuel bed were both within 15%, which is significantly lower than that of the original Byram’s method. Based on the results of this study, we propose two methods that are suitable for predicting the flame height of surface fires in the typical forests of the Yunnan–Guizhou Plateau in China, which is of great significance for further understanding the relationship between flame height, fireline intensity, and scientific forest fire management.

## Introduction

Forest fire behavior refers to a series of behavioral indicators of forest fuels after ignition, including the rate of fire spread, flame height and length, fireline intensity, and residence time^1–3^. Since forest surface fuels are the first to ignite and develop, a series of surface fire behavior indicators should be obtained to accurately predict fire ground changes and effectively command firefighting. Forest fires are more complex and variable than indoor fires because they occur in open areas and are greatly affected by fuel types, environmental factors, and terrain conditions. Obtaining accurate surface fire behavior indicators is among the important aspects of forest fire scientific research^4–6^.

Flame height is among the three indices of forest fire behavior^7^. It represents the height of a continuous flame perpendicular to the ground; the higher its value, the stronger the intensity of forest fires and the greater the damage to forest trees^8,9^. Moreover, flame height is an important indicator of the conversion of surface fires into crown fires. When the flame height exceeds the height of the forest branch, surface fires may be converted into crown fires; however, the opposite scenario is impossible^10,11^. In forest fire management, especially in understory nurturing and fire prevention forest belt planning, it is necessary to control the height of understory branches below the potential flame height of surface fires to reduce the possibility of crown fires^12–14^. Therefore, obtaining accurate flame heights is of great significance for scientific forest fire management and firefighting.

Byram proposed the forest fireline intensity model^15^, which is widely used^16^. This method established the relationship between the rate of fire spread, flame height, and forest fire intensity, and propose using fireline intensity to calculate flame height (hereinafter referred to as Method1 or M1). However, the flame height is influenced by meteorological elements, terrain conditions, fuel physicochemical properties, and fuel bed characteristics^3,17,18^, and M1 is obtained through research on specific regions and fuel types. For a specific region, the appropriateness of different fuel types for calculating flame height using M1 and the prediction accuracy of M1 need to be investigated. Research has shown that the poor applicability (large error) of predictive models is mainly due to the incomplete consideration of influencing factors or different types of fuels^19–21^. Therefore, surface fire spread experiments have been conducted to analyze the applicability of directly using M1 to estimate flame height and determine the factors influencing flame height to correct M1 or further improve the accuracy of flame height prediction.

When wind is present and the terrain is inclined, flame height is lower than flame length, thereby causing the flame to get closer to the surface, improving the heat transfer rate, and accelerating the spread of the forest fire^22^. In contrast, under flat and windless conditions, surface fires spread at a relatively slow rate, and the flames spread upward; therefore, flame height and length are similar in such cases^23^. Moreover, under similar fuel conditions, flame height is highest when there is no wind on flat ground. Furthermore, the influence of fuel bed characteristics on flame height can be better revealed when there is no wind on flat ground^21,24^; moreover, a basic model of flame height can be established, which is important for the study of flame height under wind and slope conditions in the later stages (calculated using wind and slope coefficients). Therefore, this study mainly focused on the height of surface flame under flat and windless conditions.

Located in southwest China, the Yunnan–Guizhou Plateau forest area is abundant in forest resources and species diversity. It is important for water and soil conservation, shelter forests maintenance of ecological balance in the region^25,26^. However, the forest area of the Yunnan–Guizhou Plateau has the highest number of forest fires in China, which are extremely difficult to put out, owing to its complex terrain, high mountains, steep slopes, and complex social conditions (agroforestry ecotone). Its vulnerability to forest fires has a serious impact on the environment, lives and property of people, and social stability^27^. However, the scarcity of studies on the flame height of surface fires in the typical forests in this region hinders effective forest firefighting and management. This study focuses on the surface fuel of the typical forest stands in the Yunnan–Guizhou Plateau as the research object. Specifically, we determined the factors affecting the flame height of surface fires in the typical forest stands on flat land under windless conditions and explored the feasibility of estimating the flame height of surfaces fires in the Yunnan–Guizhou Plateau based on Byram’s fireline intensity model. If the model turned out not to be applicable, we corrected the original model and provided a new flame height prediction method suitable for the study area. The findings of this study contribute to a better understanding the mechanisms of fire behavior, improving forest fire prediction accuracy, and assisting scientific forest fire management.

## Materials and method

### Sample collection

To make the results sufficient and convincing, we selected the following typical coniferous and broad-leaved forests in the Yunnan–Guizhou Plateau as research objects: *Pinus massoniana*, *Pinus yunnanensis*, *Pinus armandii*, *Quercus glauca*,* Quercus acutissima*, *Cunninghamia lanceolata*, *Phyllostachys edulis,* and *Cryptomeria japonica*. Each forest stand was set at a 25.82 m × 25.82 m standard plot. Since the fire prevention period in the Yunnan–Guizhou Plateau starts from October 1 to May 31 of the following year, forest fires frequently occur from February to April, accounting for more than 50% of forest fires per year^28^. Research has shown that the physical and chemical properties of fuels in the current and following years differ, and their impact on fire behavior is significant. Therefore, a standard site and fuel characteristics survey was conducted in February. The specific survey methods and sample site conditions can be found in the paper of Zhang and Tian^3^; meanwhile, the investigation of the *Cryptomeria japonica* forest was conducted separately (Table 1).

**Table 1:** Basic information of the sample plot and fuel characteristics of *Cryptomeria japonica.*

Slope (°)	Location	Canopy density	Mean height (m)	Mean diameter at breast height (cm)	Range of the fuel height (cm)	Range of the fuel loading (t/ha)
6	Downhill	0.81	11.36	14.82	1.82–7.30	3.84–8.66

For simplicity of writing and description, *Pinus massoniana*, *Pinus yunnanensis*, *Pinus armandii*, *Quercus glauca*, *Quercus acutissima*, *Cunninghamia lanceolata*, *Phyllostachys edulis,* and *Cryptomeria japonica* are hereafter referred to as Pm, Py, Pa, Qg, Qa, Cl, Pe, and Cj, respectively.

### Indoor burning experiment

Without considering the effects of wind and terrain, flame height is mainly related to the bed characteristics of specific fuel types. Therefore, based on the characteristics of the fuel bed in the study area, combined with the ignition and spread conditions, fuel beds with different moisture levels, loads, and height gradients were uniformly laid on the burning bed for surface fire simulation experiments. Preliminary experiments have shown that when the moisture content was 25% in the study area, only some fuels were ignited regardless of changes in bed load and height. Additionally, when the moisture content of the fuel bed on the surface of Qg was 20%, the flame height was too low to be measured. Based on this, the maximum moisture content gradient for Qg was set to 15%, while that for the other seven fuel bed layers in the indoor burning experiment was set to 20%. A total of three or four gradients were set in increments of 5%. Based on the burning situation, the bed height in this study included five gradients of 1, 2, 3, 5, and 7 cm, and the load gradients included 4, 5, 6, and 8 t/ha.

The rate of spread was required in calculating the fireline intensity in this study; therefore, the corresponding propagation speed for each flame height was recorded. The methods for laying the fuel bed layers, conducting burning experiments, and measuring the rate of spread are described in the “Burning experiment” section of the paper of Zhang and Tian^3^. The flame height was measured after the flame reached a quasi-steady state. On one side of the burning bed, a measuring rod was set every 0.2 m. When the flame head reached the measuring rod, a steel ruler (1.5 m) was used to measure the flame height. A 2-m-long burning bed had 11 measuring rods; therefore, a total of 11 flame heights were recorded in the burning experiments for this ratios. The arithmetic mean of the 11 flame heights was used as the flame height for this ratio. To avoid the influence of indoor temperature and humidity on the burning of fuels, we conducted the burning experiments in a relatively constant environment. The average temperature and relative humidity throughout the experiments were 23.8 ℃ and 65.2%, respectively.

### Statistical analysis

#### Basic statistics of flame height

The mean, median, minimum, and maximum values of the flame height of a typical stand in the Yunnan–Guizhou Plateau were analyzed under flat land and windless conditions.

#### Analysis of variance

We analyzed whether the characteristics of the surface fuel bed of different forest stands had a significant impact on flame height under flat and windless conditions and determined the specific effects of the influencing factors on flame height.

#### Applicability analysis of directly using M1

According to Byram’s model of the relationship between fireline intensity and flame height, the flame height was calculated using the following formula^15^:

$$ {\text{I}} = {\text{HWR}}/{6}00 $$

$$ {\text{F}}_{{\text{H}}} = 0.0{\text{77476I}}^{{0.{46}}} = 0.0{77476 }\left( {{\text{HWR}}/{6}00} \right)^{{0.{46}}} $$

where F_H_ represents the flame height (m), I represents the fireline intensity (kW/m), H represents the calorific value of fuels (J/g), W is the effective fuel load (t/hm^2^), R is the rate of spread (m/min), and 1/600 is the parameter for converting imperial into metric units.

For each forest stand, the mean absolute error (MAE) and mean relative error (MRE) of M1 were calculated based on the actual and predicted values using this method. A 1:1 graph was drawn using the actual values as the horizontal axis and the predicted values as the vertical axis, and analyze the error that may occur when the flame height is in which range^29^.

#### Model correction and reform

If directly using M1 in section "Applicability analysis of directly using M1" not applicable, the following steps were carried out: (1) correcting M1 to seeking to predict the relationship between flame height and fireline intensity of fuel types in the study area, and (2) reforming the flame height prediction model based on fuel bed characteristics. The discrepancies between the two method is that the first method is still based on Byram’s fireline intensity for estimation, while the second method directly uses fuelbed characteristics for estimation.

(1) Correcting M1. For each typical forest stand in the Yunnan–Guizhou Plateau, the calculated Byram’s fireline intensity and flame height were taken as the abscissa and ordinate, respectively, to draw a scatter plot. To analyze the trend of the flame height and fireline intensity, we selected the most suitable equation (such as linear, quadratic, exponential, etc*.*), obtained equation parameters using the least squares method, re-established the relationship between fireline intensity and flame height, and corrected M1, naming it as Method2 (hereinafter referred to as M2). The MAE and MRE of M2 for each forest stand were calculated.

(2) Reforming the flame height prediction model. Using fuel bed characteristics (bed moisture content, load, and height) as the independent variables and flame height as the dependent variable, linear stepwise regression was performed to reform the flame height prediction model, hereafter referred to as Method3 (hereinafter referred to as M3). The MAE and MRE of M3 for each forest stand were calculated.

The *t*-test method was performed to compare significant differences in the errors of the three methods (M1, M2, and M3) for each typical stand in the Yunnan–Guizhou Plateau.

### Statement

We confirmed that the experimental research and field studies on plants or plant parts (either cultivated or wild), including the collection of plant material were performed in accordance with the relevant guidelines/regulations/legislation.

## Results

### Basic situation statistics

**Figure 1:**
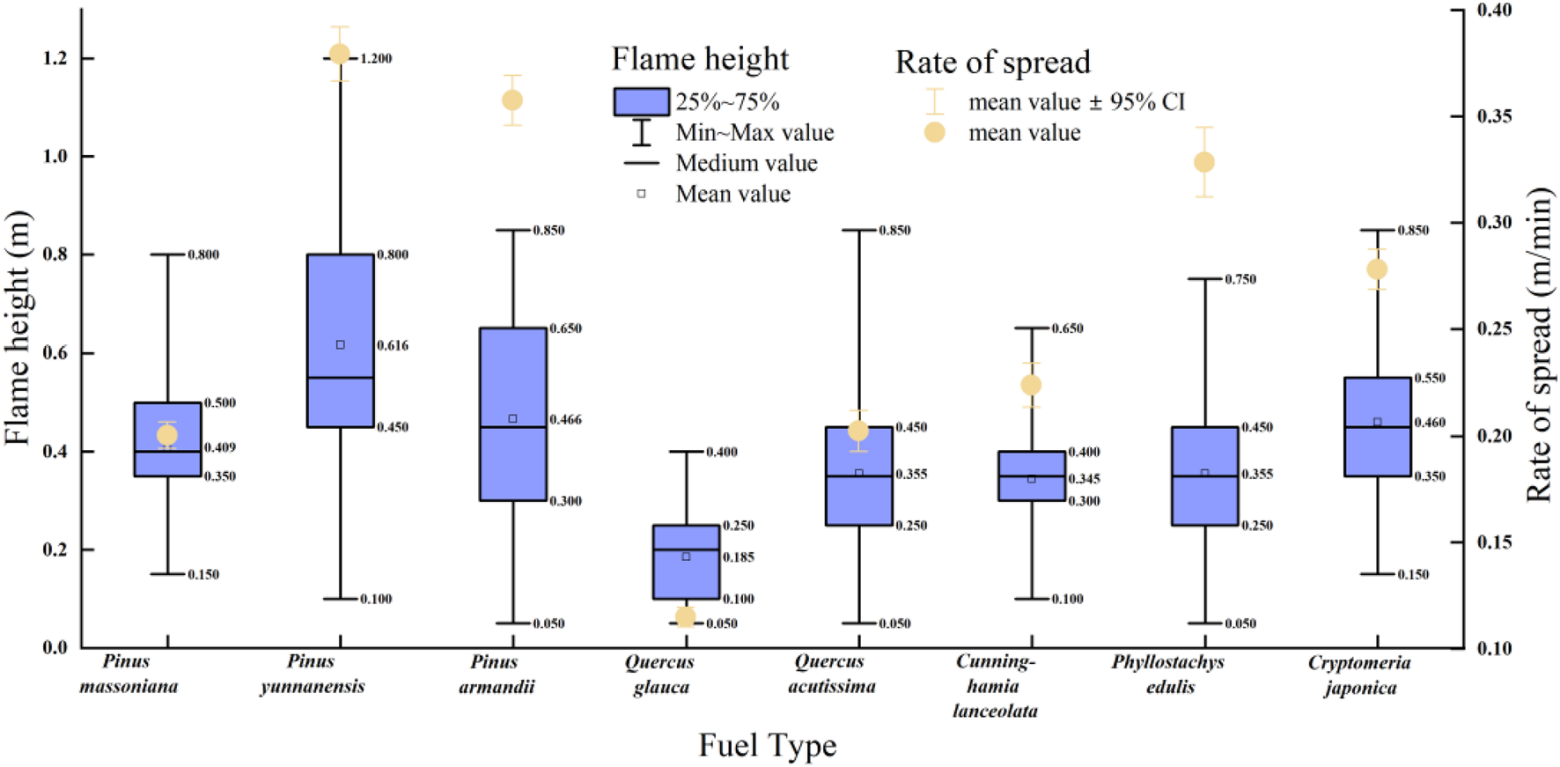
Basic situation of flame height and rate of spread.

The basic situation, including the minimum, mean, maximum, and quartile values, of the flame height and rate of spread of the typical stands in the Yunnan–Guizhou Plateau forest region is shown in Fig. 1. The maximum and minimum flame heights of surface fires in the typical forest stands in the Yunnan–Guizhou Plateau were 1.2 m and 0.05 m, respectively, under flat ground and windless conditions. The forest stands with mean flame heights in a decreasing order were Py, Pa, Cj, Pm, Pe, Qa, Cl, and Qg. Moreover, there was a correlation between the mean rate of spread and flame height.

### Analysis of influencing factors

Table 2 shows the *p*-value in variance analysis. It can be seen that the moisture content and load of surface fuels of the typical forest stands in the Yunnan–Guizhou Plateau had a significant impact on flame height (*p* value is less than 0.01). Except for Qg and Cj, the bed heights of surface fuels of the other forest stands had a significant impact on the flame height.

**Table 2:** Results of ANOVA.

Index	Fuel type							
	*Pinus massoniana*	*Pinus yunnanensis*	*Pinus armandii*	*Quercus glauca*	*Quercus acutissima*	*Cunninghamia lanceolata*	*Phyllostachys edulis*	*Cryptomeria japonica*
Moisture content	< 0.01	< 0.01	< 0.01	< 0.01	< 0.01	< 0.01	< 0.01	< 0.01
Fuel loading	< 0.01	< 0.01	< 0.01	< 0.01	< 0.01	< 0.01	< 0.01	< 0.01
Fuel bed height	< 0.01	< 0.01	< 0.01	0.161	< 0.01	< 0.01	< 0.01	0.549

The number in the table represent *p* value of the analysis of variance. When it is less than 0.05, it indicates a significant impact, and when it exceeds 0.05, it indicates no significant impact.

**Figure 2:**
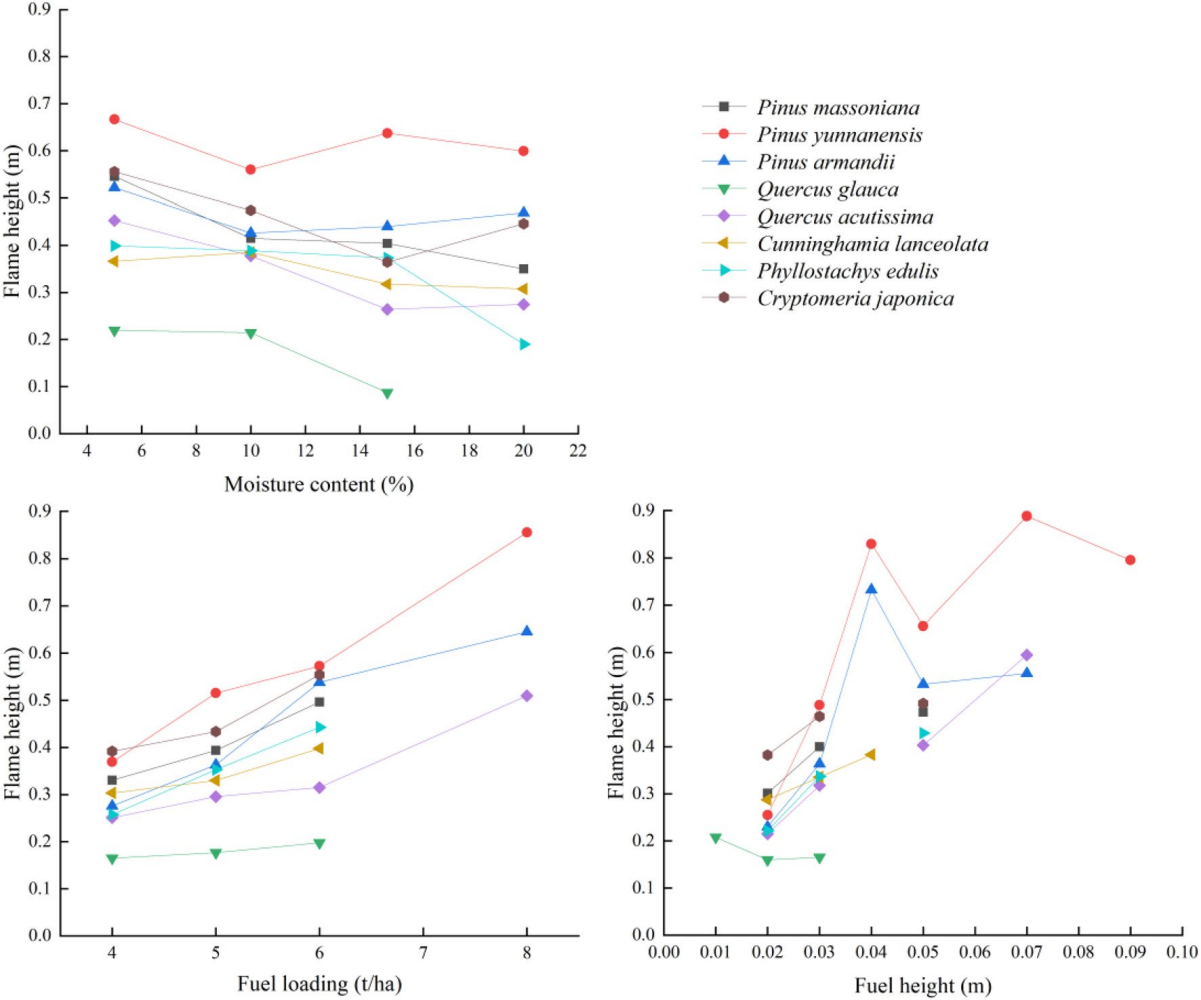
Main effect diagram of factors influencing flame height.

Figure 2 shows the main effect diagram of the factors influencing flame height. It can be seen that the flame height of the surface fuel of the typical stands in the Yunnan–Guizhou Plateau forest area decreased as the moisture content decreased. The fuel bed load had a promoting effect on the flame height; meanwhile, the fuel bed height had a promoting effect on the flame height, except in the case of Qg.

### Applicability of M1

**Figure 3:**
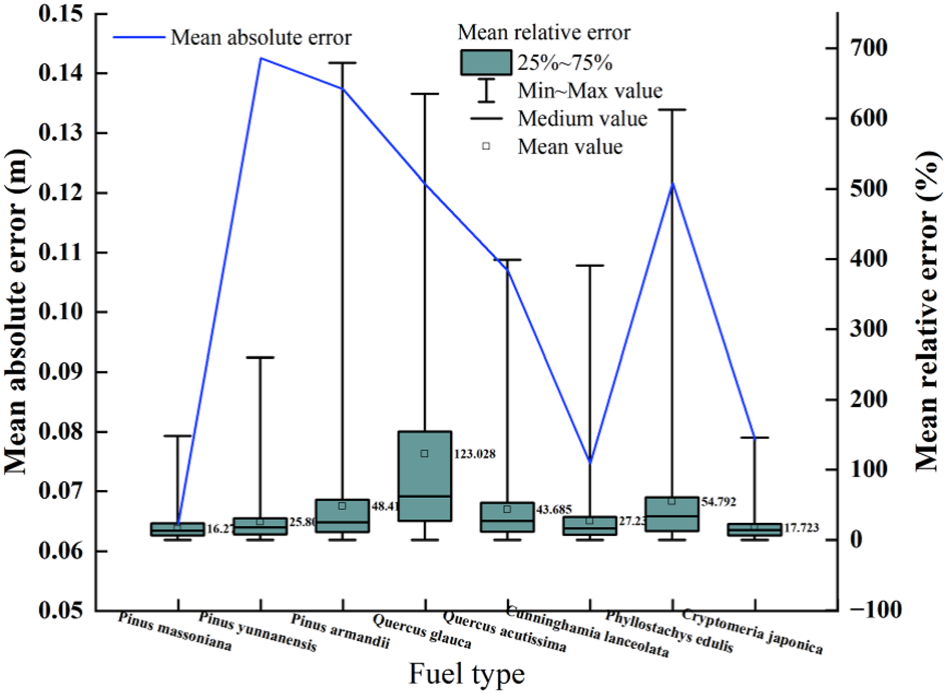
Error results of directly using M1.

The maximum value of the MRE of M1 exceeded 150%. The effect of flame height on Pm was the lowest (MRE = 16.27%), while that on Qg was the highest (MRE = 123.025%) (Fig. 3).

**Figure 4:**
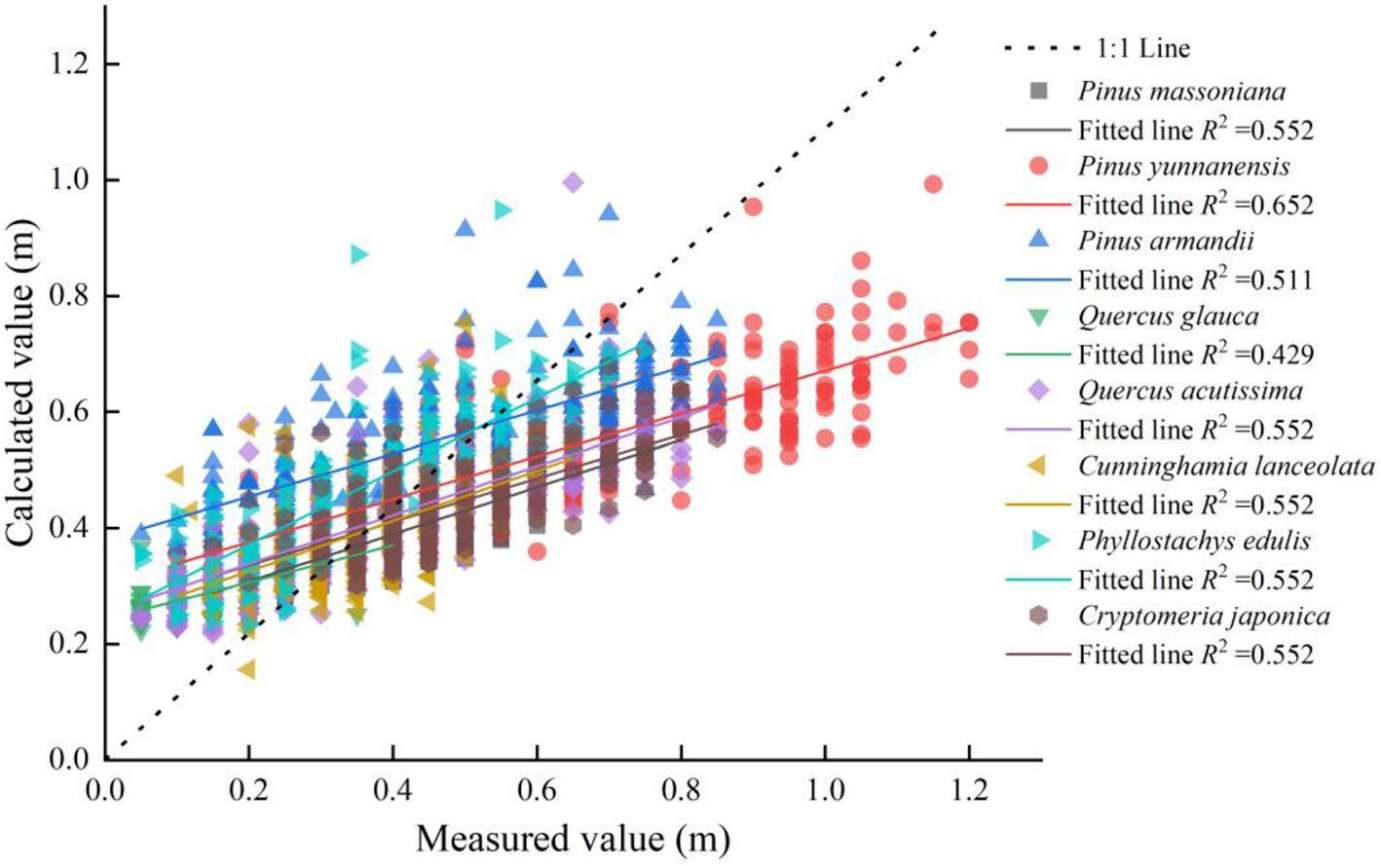
1:1 diagram of the measured and calculated values using M1.

Figure 4 is a 1:1 graph that shows that for all types of surface fuels, the flame height calculated using M1 was overestimated and then underestimated as the measured flame height increased.

### New prediction model for flame height

#### Correction of M1

**Figure 5:**
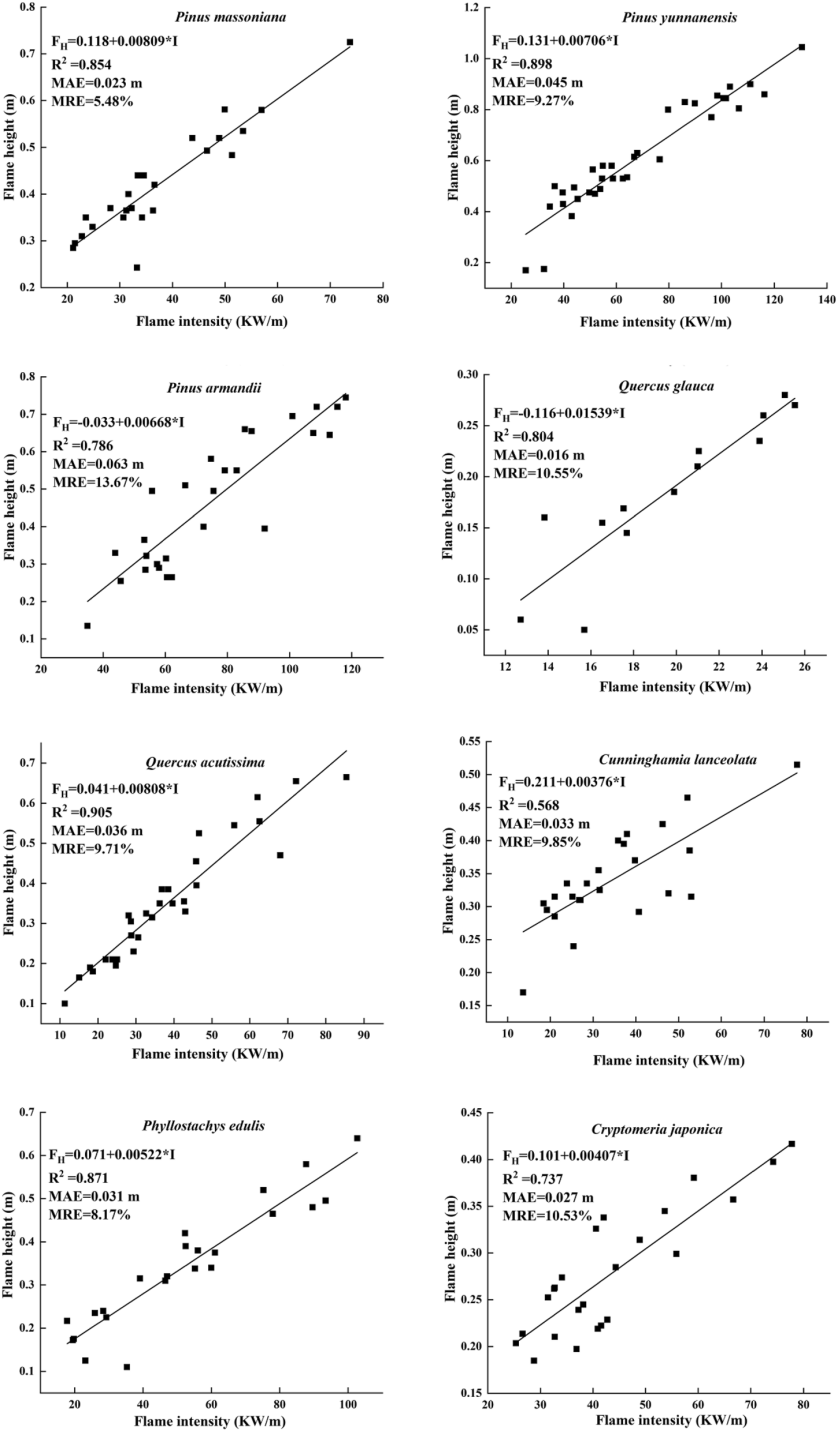
Diagram showing the relationship between fireline intensity and flame height using the M1. MAE, mean absolute error; MRE, mean relative error; F_H_, flame height; R^2^, corrected coefficient of determination.

Figure 5 shows the relationship between the flame height and fireline intensity calculated using M1. The flame height significantly increased as the fireline intensity increased. Hence, the flame height prediction model based on Byram’s fireline intensity model was corrected (M2). The MRE of M2 for the eight forest stands in the Yunnan–Guizhou Plateau forest region was less than 15%, and the minimum value was 5.48%.

#### Reforming the flame height prediction model

Table 3 shows the flame height prediction models (M3) based on the characteristics of fuel bed of the eight forests in the Yunnan–Guizhou Plateau. The fuel bed load was incorporated in all the equations. Except for Pa, the bed moisture content was considered in the flame height prediction models. The minimum MRE value of the prediction models was recorded in Pm (8.35%), while the maximum value was recorded in Qg (14.52%).

**Table 3:** Results of the flame height prediction models based on fuel bed characteristics.

Fuel type	Model	MAE (m)	MRE (%)	R^2^
*Pinus massoniana*	${F}_{H}=0.1155-1.027M+0.0663L+2.95H$	0.035	8.35	0.704
*Pinus yunnanensis*	${F}_{H}=0.002-0.788M+0.1155L$	0.066	11.10	0.738
*Pinus armandii*	${F}_{H}=-0.0864+0.0939L$	0.058	12.08	0.646
*Quercus glauca*	${F}_{H}=0.0422-1.548M+0.0527L$	0.026	14.52	0.641
*Quercus acutissima*	${F}_{H}=0.061-1.41M+0.0549L+3.572H$	0.039	11.88	0.853
*Cunninghamia lanceolata*	${F}_{H}=0.1575-0.557M+0.040L+1.56H$	0.031	8.74	0.528
*Phyllostachys edulis*	${F}_{H}=0.057-1.405M+0.0589L+4.46H$	0.055	13.89	0.528
*Cryptomeria japonica*	${F}_{H}=0.163-0.879M+0.0813L$	0.058	12.68	0.455

MAE, mean absolute error; MRE, mean relative error, R^2^, corrected coefficient of determination. $M$, $L$, $H$, represent the fuelbed moisture content (%), fuelbed load (t/ha) and fuelbed height (m), respectively.

### Comparison of the methods

**Figure 6:**
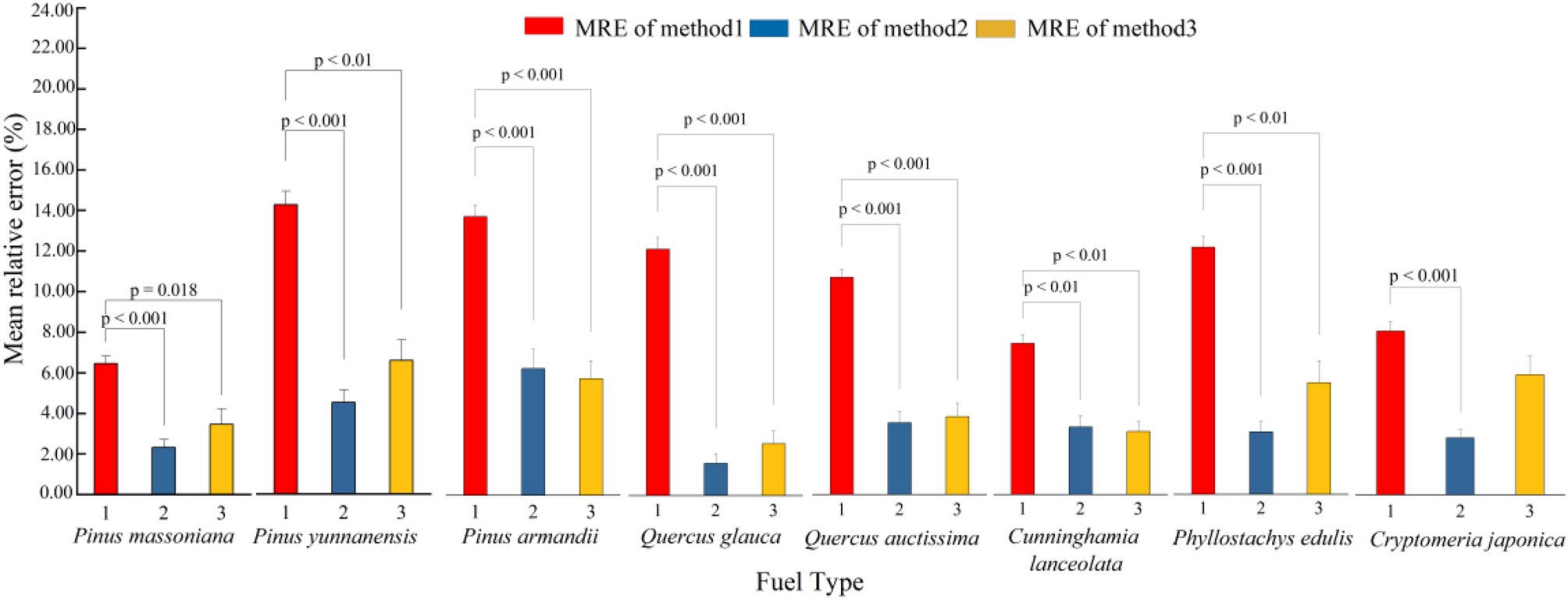
Comparison of the mean absolute errors of M1, M2, and M3.

Figure 6 shows the differences among the MREs of the three methods for predicting the height of surface flames in the typical stands in the Yunnan–Guizhou Plateau. Except for Cl and Cj, the MREs of M2 and M3 for the other forest stands were lower than those of M1; however, there was no significant difference in MRE between M2 and M3.

## Discussion

### Basic information of flame height

Within the scope of this study, the surface flame height of fuels in the typical forests of the Yunnan–Guizhou Plateau ranged from 0.05 to 1.20 m. The flame height of surface fires in forests is generally 0.50–1.50 m or 0.37–2.50 m according to Niu^30^ and Wang et al*.*^31^, respectively. The minimum value of the flame height recorded in this study was low, probably due to different fuel types and bed characteristics. Liu conducted indoor simulation burning under flat and windless conditions with a maximum flame height not exceeding 0.69 m in the coniferous bed of a typical pine forest in Northeast China^32^, which is within the scope of this study. The mean flame height of Qg was the lowest at 0.181 m, while that of Py was the highest at 0.616 m. The differences in the flame height of surface fires among the different forest stands were mainly due to different types of fuels. The different physical and chemical properties of fuel types, including surface area-to-volume ratio, calorific value, and mineral content, significantly affect surface fire behavior^3^.

### Analysis of the factors affecting flame height

Under flat and windless conditions, the flame heights of the surface fires in Qg and Cj were only significantly correlated with the bed moisture content and load. For the other stands, the fuel bed moisture content, load, and bed height had a significant impact on the flame height, which is similar to the results of Chu^26^. The flame heights of Qg and Cj were not correlated with the bed height, probably due to the gradient setting of the bed heights of these two fuel types. In this study, the surface bed heights of the two fuel types were approximately 0.01–0.03 m and 0.02–0.05 m, respectively, with little difference in gradient. Therefore, the influence of bed height on flame height was not observed.

In this study, the flame height decreased as the bed moisture content increased. The bed moisture content represents the amount of moisture in the fuel, which determines the evolution and intensity of fire behavior after a fire occurs^33^. The higher the moisture content of the fuel, the more energy is needed to evaporate water; as the energy used for combustion decreases, the flame height decreases^34^. The bed load represents the absolute dry mass of fuel per unit area; the larger the load, the higher the energy released per unit area after the ignition of fuels. Therefore, as observed in this study, the flame height increased as the load increased (Fig. 2). However, as the bed height increased, the flame height fluctuated between different gradients although it exhibited an overall increasing trend because of the possible interaction between the bed load and height. Because the burning bed area in this study was fixed at 2 m^2^, the interaction between the bed load and height can be understood as the influence of bed density on flame height. Although a larger bed density can increase heat accumulation and promote combustion, an excessively high density will reduce the combustibility of oxygen and other materials in the combustion zone and, thus, inhibit combustion. Therefore, fuel bed density has a dual effect on flame height^29,35^; meanwhile, the effect of bed height on flame height is influenced by the bed loa^d^^26,32^, resulting in flame height fluctuations between different gradients.

### Applicability analysis of directly using Byram’s fireline intensity model to estimate flame height

The maximum relative error of the flame heights of the surface fuels in the Yunnan–Guizhou Plateau exceeded 150% under windless and flat conditions, and the flame height prediction error for Pm was the lowest (16.27%) because flame height is significantly related to the physical, chemical, and bed characteristics of fuel. Although fireline intensity fully considers the rate of spread, calorific value, and load, the response of flame height to the physical, chemical, and bed properties of different fuels is different. An unreasonable setting of model parameters will inevitably lead to the inability to use them directly, which is similar to the results of Albini^36^ and Sullivan^37^. The high error further confirmed that although the estimation of flame height based on Byram’s fireline intensity model has a certain universality and can reflect the flame height situation to a certain extent, the model needs to be further corrected for different fuel types. Therefore, directly using M1 to estimate the flame height of typical stand fires in the Yunnan–Guizhou Plateau is not appropriate, and it is necessary to correct or reform the prediction model.

### Correcting and reforming a new prediction model

The flame height of the surface fuels of the typical forests in the Yunnan–Guizhou Plateau was significantly linearly related to the fireline intensity under flat ground and windless conditions. Under different test conditions and variables, Fernandes et al*.* took into account wind speed and slope; they found out that the flame height was less than the flame length^38^. Therefore, they concluded that the flame height changed exponentially with the fireline intensity. However, on flat land without wind, the flame did not tilt forward, and its height was equal to its length. In this case, the flame height (length) increased linearly with the fireline intensity. Therefore, M1 was corrected, and the MRE of M2 for the eight fuel types in the Yunnan–Guizhou Plateau region was lower than 15%, while the minimum value was only 5.48%. Moreover, the prediction error of M2 was significantly lower than that of directly using M1; hence, it can be applied in practice.

Due to different calculation principles, there are apparent discrepancies between M3 and M2. Whether by directly using or M2, flame height is estimated by inputting factors such as the rate of spread and physicochemical properties of the fuel after a forest fire occurs. However, when conducting planned burning, creating biological fire prevention forest belts, nurturing under the forest, or judging forest fire intensity in the early stages of a fire, it is necessary to estimate the flame height or calculate the forest fire intensity based on the flame height^9^. Therefore, a flame height prediction model for surface fires in the typical forests in the Yunnan–Guizhou Plateau was also established in this study based on the fuel bed characteristics (M3). Liu established a flame height prediction model for surface fires in Korean pine and Mongolian oak forests based on bed characteristics, and the additive model had the best prediction effect^32^. The results of the study of Liu were consistent with our results, and an additive prediction model was established based on the characteristics of the fuel bed. The bed load was entered into the equation; however, except for Pa, all the other models for predicting the flame height of the fuel bed considered the bed moisture content. The maximum MRE value of all the models was only 14.52%, which was within the allowable error range. It is generally believed that when the MRE is below 15%, it meets the practical application requirements^39,40^. Some equations do not take into account the bed moisture content and height, while the analysis of vairance has a significant impact. This is mainly because this study chose linear stepwise regression as the modeling method, which will more accurately obtain the relationship between the influencing factors and the flame height while excluding other related factors during the modeling process, ensuring the maximum R^2^ and minimum error. The error of M3 was not significantly different from that of M2; since its error was lower than that of M1, M3 can be directly used in practice.

### Forest fire management

The maximum and mean flame heights of the surface fuels in the typical stands of the Yunnan-Guizhou Plateau were 1.20 m and 0.53 m, respectively, under flat ground and windless conditions. However, in the presence of an airflow or slope, the flame height will decrease. Therefore, to prevent surface fires from spreading upwards into canopy fires, it is necessary to ensure that the height of the understory branches in these stands is controlled below the flame height when conducting understory pruning, cleaning, and fire prevention forest belt planning. A flame height prediction model based on fuel bed characteristics, without relying on the rate of spread and fireline intensity after a fire occurs, was developed in this study. In forest fire management, the flame height of a forest fire can be estimated based on the existing fuel bed characteristics, and work can be carried out accordingly.

The main purpose of this study was to analyze the impact of fuel bed on the flame height of a ground fire, test the applicability of Byram’s fireline intensity model to estimate the flame height in typical forests in the Yunnan–Guizhou Plateau, and correct the model. Therefore, the flame height measurement experiment was conducted only under flat and windless conditions without considering the effects of wind speed and terrain, which limited the scope of this study. However, it still provides basic data and methods for studying flame height. Moreover, this study was conducted indoors and the structure of the fuel bed differed from the actual structure in the field. Therefore, in future research, it is necessary to increase the number of field validation tests, consider wind speed and terrain factors, and establish a more comprehensive flame height prediction model for the typical forests in the Yunnan–Guizhou Plateau. In addition, fuel bed characteristics were used as the independent variables and a flame height prediction model was established through linear stepwise regression, which to some extent concealed the actual influence of features on flame height. Thus, in future research, it is necessary to peel of the individual impact of each bed characteristics on flame height, and establish a flame height prediction model through model coupling to further improve the prediction accuracy of the model. At the same time, it is necessary to utilize existing modified models based on Byram and conduct comparative studies of different models in order to obtain the most suitable prediction model.

## Conclusion

Directly using Byram’s fireline intensity model was not applicable for calculating the flame height of the typical stands in the Yunnan–Guizhou Plateau under flat land and windless conditions. The uncertainty of the physical, chemical, and bed characteristics of fuels led to a four-fold error. The surface flame height of the typical forest stands in the Yunnan–Guizhou Plateau ranged from 0.05 to 1.20 m under flat and windless conditions. Therefore, when conducting understory pruning and fire prevention forest belt planning, it should be ensured that the height under branches is less than 1.2 m. Moreover, the flame height of almost all the stands decreased significantly as the bed moisture content increased and exhibited a significant positive correlation with bed load and height. After correcting Byram’s fireline intensity model, a new fire height prediction model established based on fuel bed characteristics had a maximum error of 13.67%, which was within the allowable error range. This model is simple, easy to use, and applicable in practice. However, appropriate model applications should be selected based on their actual use in forest fire management. For example, in the event of a fire, the actual fireline intensity can be calculated based on M2 and the observed flame height, and when forest management and nurturing needed, the potential maximum flame height can be directly calculated based on the M3 to determine the height of the branches.

### Supplementary Information

Supplementary Information.

## Data Availability

Data associated with this research are available and can be obtained by contacting the corresponding author upon reasonable request.

## Abbreviation

MAE: Mean absolute error
MRE: Mean relative error
